# Transcriptome and metabolome analyses reveal the key genes related to grain size of big grain mutant in Tartary Buckwheat (*Fagopyrum tartaricum*)

**DOI:** 10.3389/fpls.2022.1079212

**Published:** 2022-12-22

**Authors:** Xiaomei Fang, Yingqian Wang, Jingbin Cui, Linqing Yue, Aohua Jiang, Jiaqi Liu, Yichao Wu, Xingxing He, Chunhua Li, Jian Zhang, Mengqi Ding, Zelin Yi

**Affiliations:** ^1^ College of Agronomy and Biotechnology, Southwest University, Chongqing, China; ^2^ Engineering Research Center of South Upland Agriculture, Ministry of Education, Chongqing, China; ^3^ Baicheng Academy of Agricultural Sciences of Jilin Province, Baicheng, Jilin, China

**Keywords:** Tartary buckwheat, grain size, transcriptome analysis, WGCNA, metabolome

## Abstract

Grain size with high heritability and stability is an important selection target during Tartary buckwheat breeding. However, the mechanisms that regulate Tartary buckwheat grain development are unknown. We generated transcriptome and metabolome sequencing from 10 and 15 days past anthesis (DPA) grains of big grain mutant (*bg1*) and WT, and identified 4108 differentially expressed genes (DEGs) including 93 significantly up-regulated differential genes and 85 significantly down-regulated genes in both stages, simultaneously. Meanwhile, we identified DEGs involved in ubiquitin-proteasome pathway, HAI-KU (IKU) pathway, mitogen-activated protein kinase (MAPK) signaling pathway, plant hormone (auxin, brassinosteroids and cytokinins) transduction pathway and five transcription factor families, including APETALA (AP2), GROWTH-REGULATING FACTORS (GRF), AUXIN RESPONSE FACTOR (ARF), WRKY and MYB. Weighted gene co-expression network analysis (WGCNA) was performed and obtained 9 core DEGs. Conjoint analyses of transcriptome and metabolome sequencing screened out 394 DEGs. Using a combined comprehensive analysis, we identified 24 potential candidate genes that encode E3 ubiquitin-protein ligase HIP1, EMBRYO-DEFECTIVE (EMB) protein, receptor-like protein kinase FERONIA (FER), kinesin-4 protein SRG1, and so on, which may be associated with the big-grain mutant *bg1*. Finally, a quantitative real-time Polymerase Chain Reaction (qRT-PCR) assay was conducted to validate the identified DEGs. Our results provide additional knowledge for identification and functions of causal candidate genes responsible for the variation in grain size and will be an invaluable resource for the genetic dissection of Tartary buckwheat high-yield molecular breeding.

## Introduction

Tartary buckwheat (*Fagopyrum tartaricum* Gaertn; 2n = 2x = 16) belongs to the Polygonaceae family and *Fagopyrum* genus, and is a dicotyledonous minor crop with homostylous and self-pollinated (Bonafaccia and Fabjan, 2003). The cultivated Tartary buckwheat, which originated in southwestern China (Ohnishi, 1998; Ohnishi and Konishi, 2001), had strong adaptability and high resistance to various environmental stressors (Wang et al., 2015). Tartary buckwheat grain is rich in vitamins, minerals, protein, dietary fiber and much higher levels of antioxidants, such as rutin, which have the functions of reducing cholesterol levels, blood clots and high blood pressure (Middleton et al., 2000; Panwar et al., 2012; Suzuki et al., 2014a; Nishimura et al., 2016).

Grain size, the combination of grain length, grain width and grain thickness, is one of the important quantitative characteristics affecting yield in Tartary buckwheat, which had high heritability and stability (Li et al., 2019). Therefore, the molecular mechanism of grain size/weight are pivotal for increasing yield of Tartary buckwheat. Previous studies had shown that the development of grain size was dependent on multiple pathways. *SHORT HYPOCOTYL UNDER BLUE 1* (*SHB1*), *HAI-KU1* (*IKU1*), *IKU2* and *MINI-SEED 3* (*MINI3*) function in the IKU pathway to control grain size by influencing endosperm growth (Luo et al., 2005; Zhou et al., 2009; Wang et al., 2010; Kang et al., 2013; Cheng et al., 2014). The ubiquitin receptor *DA1*, DA1-related protein (*DAR1*), E3 ubiquitin ligases *DA2* and BIG BROTHER (*BB*)/ENHANCER OF DA1 (*EOD1*) involved in the ubiquitin-proteasome pathway have been shown to influence grain size (Li et al., 2008; Xia et al., 2013; Du et al., 2014). The mitogen-activated protein kinases (MAPK) pathway contains three cascade reactions, which played an important role in the regulation of grain size (Duan et al., 2014; Liu et al., 2015a; Guo et al., 2018; Liu et al., 2018). Several phytohormones such as brassinosteroids (Brs) (Mora-García et al., 2004; Li et al., 2011; Xu et al., 2015), auxins (IAA) (Mizukami and Fischer, 2000; Schruff et al., 2006; Meng et al., 2015; Liu et al., 2015b) and cytokinins (CTKs) (Riefler et al., 2006; Hutchison et al., 2006) have been suggested to play an important role in grain growth. Furthermore, transcription factors played important roles in controlling grain size by either enhancing or repressing cell division and expansion, such as APETALA2 (AP2) (Johnson et al., 2002; Ohto et al., 2005; Ohto et al., 2009), AP2-like (Mizukami and Fischer, 2000), WRKY (TTG2) (Johnson et al., 2002), AUXIN RESPONSE FACTOR (ARF) (Adamski et al., 2009; Zhang et al., 2015) and growth regulating factors (GRF) (Kim and Kende, 2004; Hu et al., 2015; Duan et al., 2015).

In Taratry buckwheat, Huang et al. (2017) performed a comprehensive global transcriptome analysis using rice Tartary buckwheat grains at different development stages, namely pre-filling stage, filling stage, and mature stage, and identified 108 specifically expressed genes, and identified 11 676 DEGs, including 633 DEGs related to plant hormones, 10, 20 and 30 DEGs involved in the biosynthesis of grain storage proteins, flavonoids and starch. Liu et al. (2018) analyzed the fruit development of two species of Tartary buckwheat and indicated that the balance of AUX and ABA might be the key factor that regulated the cell division rate, which influenced the final fruit size. *FtARF2* and *FtSAURs* regulated the fruit size through hormone signaling pathway in Tartary buckwheat (Liu et al., 2018). Li et al. (2021) performed small RNA (sRNA) sequencing for Tartary buckwheat grains at three developmental stages and identified 76 miRNAs exhibited differential expression during grain development. The transcriptional dynamics of Tartary buckwheat grains at three developmental stages was assessed and 4249 DEGs, including 88 phytohormone biosynthesis signaling genes, 309 TFs, 16 expansin genes participating in cell enlargement, and 37 structural genes involved in starch biosynthesis were candidate key grain development genes (Jiang et al., 2022). Besides, phytohormone ABA, AUX, ET, BR and CTK, and related TFs could substantially regulate grain development in Tartary buckwheat through targeting downstream expansin genes and structural starch biosynthetic genes (Jiang et al., 2022). However, the molecular mechanism affecting grain size in Tartary buckwheat is still unclear.

The tiny flowers (~2 mm) and strict self-pollination of Tartary buckwheat make it difficult to construct segregation populations and map-based cloning of key genes controlling grain size by forwarding genetic approaches (Zhang et al., 2017). Transcriptome and metabolome association analysis provides an effective thought to explore key candidate genes controlling grain size of Tartary buckwheat. In this study, the mutant *bg1* with big-grain was obtained by EMS mutagenesis of cv. Pinku 1, Tartary buckwheat sequencing variety (Zhang et al., 2017). Transcriptome, metabolome and their combined analysis were carried out in grains at two development stages of *bg1* and WT (cv. Pinku 1). Combined with the functional gene analysis, DEGs analysis involved in signaling pathways of grain size, weighted gene co-expression network analysis (WGCNA) and conjoint analyses of transcriptome and metabolome sequencing were performed to explore key regulatory factors and functional genes regulating the grain size and quality of Tartary buckwheat, and understand the development mechanism of Tartary buckwheat yield and quality, so as to provide valuable insights into yield and quality in Tartary buckwheat.

## Materials and methods

### Plant materials

The cultivar Pinku 1 (WT line) was chosen for construction of the mutant library (Xu et al., 2015). Four thousand seeds were soaked in clean water (12h), and then soaked in 0.6% ethyl methane sulphonate (EMS) solution overnight (12 h). Finally, 0.1 mol/L sodium thiosulfate solution and clean water successively rinsed seeds for 5 min (repeated 3–5 times, respectively). The seeds were subsequently air-dried under a fume hood and then immediately sown in the field. In 2019 spring, M1 seeds were harvested and then the seeds selected individual lines from M2- M5 were planted at the Southwest University experimental station during the spring and autumn from 2019 to 2021. In 2021 spring, a genetically stable big-grain mutant line, *bg1*, was obtained. Afterward, *bg1* and WT lines were grown in the field and all the agronomic trait data were measured in 2021 spring.

Mature grains were harvested by hand for measurements of hundred-grain weight (HGW), grain length (GL), grain width (GW), and ratio of length-width (RLW). Flowers in full bloom were selected for lanyard marking. Grains of *bg1* and WT at 5, 10, 15, 20, 25 and 30 days post-anthesis (DPA) were taken for statistical analysis of grain development, and 10-grain length, 10-grain width and hundred-grain weight were measured in three biological replicates, which had been used to convert to grain length and grain width for reducing errors.

### Measurement of quality traits

The quality of *bg1* and WT grains was analyzed with 10 biological replicates. Flavonoid was isolated using the method of Zhang et al. (2021). The absorbance of flavonoid sollution was measured with a microplate reader at a wavelength of 420 nm. Rutin standard curve was drawn to obtain the concentration of total flavonoids, and the percentage content of total flavonoids (X%) was calculated according to the absorbance value (C), volume (V), dilution ratio (N/mL) and sample mass (M/g), X%=(C×N×V/M×10^6^)×100%.

Buckwheat starch was isolated using a modified method of Huang et al. (2007). The contents of starches was determined according to a modified version of GB/T 15683-2008/ISO 6647-1 (2008).

Protein was isolated using the sequential extraction method (Padhye and Salunkhe, 1979). Protein component content were determined by Brandford method (Walker et al., 2002). The standard curves of each protein component were drawn, OD value was detected by spectrophotometer and the content of each protein component was calculated.

### Transcriptome sequencing and differential expression analysis

Based on the morphology and statistical analysis of grain development, grains of *bg1* and WT were collected at 10 and 15 days past anthesis (DPA) for RNA extraction using TRIzol reagent (Invitrogen, USA) with three individual biological replications and then the transcriptomic experiments were conducted by BMKcloud, Beijing, China (http://www.biomarker.com.cn) following the manufacturer’s instructions. Clean reads were obtained by removing adapters, reads containing poly-N and low-quality reads, were then mapped to the Tartary buckwheat genome (download from www.mbkbase.org/Pinku1) using Hisat2 (Pertea et al., 2016), and the gene expression levels were quantified with HTseq (Anders, 2010).

Differential expression analysis of two samples was performed using the DEGseq (2010) R package under the following standard parameters: false discovery rate (FDR) < 0.05 and |log2(ratio)| ≥ 1. The GO term and KEGG pathway analysis results were considered significant when the Bonferroni (Q-value)-corrected p-value was ≤0.05. The enriched KEGG pathways were determined using R software, which was also used to construct scatter diagrams of the results. Furthermore, some key DEGs associated with grain size according to the previous research were used to construct a heatmap.

### Weighted gene co-expression network analysis

WGCNA software package (version 1.6.6) in R program was used to construct weighted gene co-expression networks for *bg1* and WT. After entering the normalized gene expression matrix, pickSoftThreashold in the WGCNA package was used to calculate the weighted value. The blockwiseModules were used to construct scale-free networks, with default parameters. The softconnectivity function was used to calculate the connectivity degree of genes for obtaining the expression modules. Correlations analysis between expression modules and yield-related traits (Hundred -grain weight, grain length, grain width) and quality (flavonoid and protein fraction) were carried out to screen the specificity module. Cytoscape (version 3.9.1) was used to visualize the network in the module (Su et al., 2014) and screen out the core genes. Core genes are selected based on the correlation between the gene and other genes or their position in the regulatory network. If the correlation between the gene and more genes exceeds the threshold, or the node position of the gene in the regulatory network, then the gene is the core gene, and the threshold value of correlation coefficient r > 0.95.

### Metabolome sequencing analysis

Samples were collected at 10 and 15 DPA grains of *bg1* and WT for metabolome sequencing analysis, which were consisitenti with samples used in the RNA-seq analysis. Samples were ground to powder using a grinder (MM 400, Retsch) and dissolved into extraction solution to extract by ultrasonic extraction. The extracted metabolites were analyzed by liquid chromatography tandem mass spectrometry (LC-MS/MS) with Waters Xevo G2-XS QTOF. The metabolomic experiments and conjoint Analyses of transcriptome and metabolome sequencing were conducted by BMKcloud, Beijing, China (http://www.biomarker.com.cn/) following the manufacturer’s instructions.

### qRT-PCR validation of DEGs

After RNA-seq, qRT-PCR was performed by SYBR Premix Ex Taq II (Tli RNaseH Plus) in a volume of 10 μL, which contained 5 μL of SYBR Green Master Mix, 200 ng cDNA template, and 0.5 μM of each of the forward and reverse primers. The qRT-PCR amplification conditions were as follows: 95°C for 30 s, followed by 40 cycles at 95°C for 5 s and 60°C for 30 s in Bio-Rad CFX manager 2.0. The relative expression levels were estimated from the threshold of PCR cycle with 2–ΔΔCt method (Livak and Schmittgen, 2001). The values from three independent biological replicates and three technical replicates were averaged.

### Statistical analysis

Results were shown as means ± SD (n = 3). Multiple testing corrections were carried out to identify the difference of values using SPSS version 21.0 (SPSS, Chicago, USA), and P < 0.05 and P < 0.01 were considered statistically significant. The heatmaps were created based on Log10 FPKM of DEGs between WT and *bg1*. Pearson’s correlation coefficient is the most commonly used method for calculating the relationship between FPKM values of candidate genes and yield and quality-related traits of mutant *bg1* and WT.

## Results

### Analysis of phenotypic characteristics

**Figure 1:**
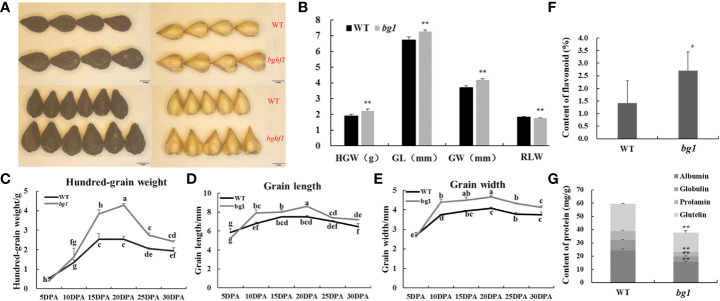
Morphological observations and statistical analysis of grain of WT and mutant bg1. **(A)** Morphological observations of grain at mature period. **(B)** Statistical analysis of grain traits at mature period. **(C, D)** Statistical analysis of grains at different development stages in WT and *bg1*. **(F, G)** Statistical analysis of content of flavonoid and storage protein components The scale bar = 2 mm in A. *P<0.05, **P<0.01. The same letter are not significantly different using multiple comparisons (P < 0.05) in **(C–E)**.

Compared with WT, the hundred-grain weight (HGW) of *bg1* was increased by17.55%, the grain length (GL) increased by 8.49%, the grain width (GW) increased by 13.55%, and the ratio of length to width decreased by 4.92 (p < 0.01 for each trait) (**Figures 1A, B**). Statistical analysis were carried out on the grains of *bg1* and WT at 5, 10, 15, 20, 25 and 30 DPA (**Figures 1C–E**). Hundred-grain weight, grain length and grain width of WT and *bg1* increased gradually with the growth process, and reached the maximum at 20 days, and then decreased slightly due to dehydration and maturation. Hundred-grain weight, grain length and grain width of *bg1* were higher than those of WT after 5 DPA (**Figures 1C–E**).

For quality traits, the flavonoid content of *bg1* (2.7%) was significantly higher than that of WT (1.4%) and the content of storage protein components were significantly lower than that of WT (**Figures 1F, G**).

### Transcriptome sequencing and analysis of DEGs in Tartary Buckwheat

**Figure 2:**
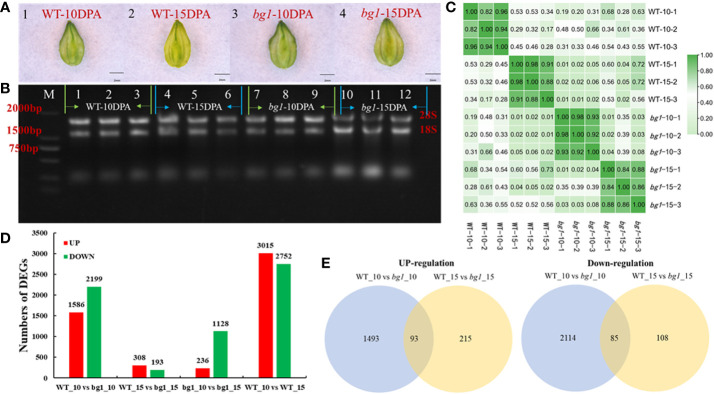
Transcriptome sequencing and analysis of DEGs in Tartary Buckwheat. **(A)** Morphological observation of sampled grains. **(B)** Results of agarose electrophoresis of total RNA from samples. **(C)** Correlation analysis between samples. **(D)** The number of up-and down-regulated differentially expressed genes in different comparisons. **(E)** Venn diagram of differentially expressed genes.

To investigate the underlying mechanisms that control grain size of Tartary buckwheat, according to the statistical analysis of grain development (**Figure 1**), high-throughput RNA-Seq was performed at 10 DPA and 15 DPA grains of WT and *bg1*, respectively, with three biological replicates for each sample (**Figures 2A, B**). The correlation evaluation of biological replicates showed a high correlation (**Figure 2C**). A total of 12 libraries were constructed and sequenced using the Illumina HiSeqTM 4000 platform. A total of 84.46 Gb of Clean Data was obtained from 12 samples. The percentage of Q30 bases in each sample ranged from 92.61% to 93.62%, and the GC content ranged from 45.86% to 48.23% (**Supplementary Table 1**).

Transcriptome sequencing showed that 3785 differentially expressed genes were obtained in WT_10 vs *bg1*_10 comparison, including 1586 up-regulated genes and 2199 down-regulated genes. A total of 501 differentially expressed genes were obtained in WT_15 vs *bg1*_15 comparison, including 308 up-regulated genes and 193 down-regulated genes, with 93 significantly up-regulated differential genes and 85 significantly down-regulated genes in both stages, simultaneously (**Figures 2D, E**; **Supplementary Table 2**).

The GO enrichment analysis of DEGs was performed between WT and *bg1* (**Table 1**). The up-regulated DEGs were enriched in plasma membrane (GO:0005886) and multicellular organism development (GO:0007275), multicellular organismal processes (GO:0032501), gametophyte development (GO:0048229) and pollen development (GO:0009555). The down-regulated DEGs were significantly enriched in nucleus (GO:0005634), regulation of cellular process (GO:0050794), macromolecular complex (GO:0032991).

**Table 1:** GO enrichment of differentially expressed genes in two periods.

	GO classify1	GO_ID	Term	DEGs	P-value
Up	cellular component	GO:0005886	plasma membrane	13	0.010194
	biological process	GO:0007275	multicellular organism development	11	0.002327
	biological process	GO:0032501	multicellular organismal process	11	0.003291
	biological process	GO:0048229	gametophyte development	6	0.000232
	biological process	GO:0009555	pollen development	5	0.000968
Down	cellular component	GO:0005634	nucleus	17	0.016746
	biological process	GO:0050794	regulation of cellular process	9	0.013897
	cellular component	GO:0032991	macromolecular complex	5	0.02336

To further understand the metabolic pathways in which DEGs were involved, KEGG analyses were performed (**Table 2**). The up-regulated DEGs in both periods were highly significantly enriched in Tyrosine metabolism (KO00350), arginine and proline metabolism (KO00330), selenocompounds metabolism (KO00450), glycolysis/gluconeogenesis (KO00010), brassinosteroid biosynthesis (KO00905), glyoxylate and dicarboxylate metabolism (KO00630), glycosphingolipid biosynthesis-ganglio series (KO00604), glycine, serine and threonine metabolism (KO00260), carotenoid biosynthesis (KO00906) and citrate cycle (KO00020). Down-regulated DEGs were highly significantly enriched in ABC transporters (KO02010), plant hormone signal transduction (KO04075) and betalain biosynthesis (KO00965), cysteine and methionine metabolism (KO00270), flavonoid and flavonol biosynthesis (KO00944), diterpenoid biosynthesis (KO00904) and monoterpenoid biosynthesis (KO00902), tyrosine metabolism (KO00350), fatty acid biosynthesis (KO00061), protein processing in endoplasmic reticulum (KO04141), benzoxazinoid biosynthesis (KO00402), MAPK signaling pathway plant (KO04016), cutin, suberine and wax biosynthesis (KO00073).

**Table 2:** KEGG enrichment analysis of differentially expressed genes.

	Ko ID	Pathway	Rich factor	q-value	Selected	All Gene
Up	ko00350	Tyrosine metabolism	0.904	0	4	80
	ko00330	Arginine and proline metabolism	1.39	0	8	104
	ko00450	Selenocompound metabolism	3.389	0	6	32
	ko00010	Glycolysis/Gluconeogenesis	1.359	0.001	17	226
	ko00905	Brassinosteroid biosynthesis	1.643	0.002	2	22
	ko00630	Glyoxylate and dicarboxylate metabolism	1.1	0.002	7	115
	ko00604	Glycosphingolipid biosynthesis-ganglio series	1.13	0.007	3	48
	ko00260	Glycine, serine and threonine metabolism	0.853	0.007	5	106
	ko00906	Carotenoid biosynthesis	0.488	0.008	2	74
	ko00020	Citrate cycle (TCA cycle)	0.415	0.009	2	87
Down	ko02010	ABC transporters	2.317	0	29	175
	ko04075	Plant hormone signal transduction	1.512	0	85	786
	ko00965	Betalain biosynthesis	4.495	0	9	28
	ko00270	Cysteine and methionine metabolism	2.177	0	26	167
	ko00944	Flavone and flavonol biosynthesis	5.993	0	6	14
	ko00904	Diterpenoid biosynthesis	3.108	0	12	54
	ko00902	Monoterpenoid biosynthesis	2.844	0.001	12	59
	ko00350	Tyrosine metabolism	2.447	0.001	14	80
	ko00061	Fatty acid biosynthesis	2.359	0.002	14	83
	ko04141	Protein processing in endoplasmic reticulum	1.528	0.002	46	421
	ko00402	Benzoxazinoid biosynthesis	3.814	0.004	6	22
	ko04016	MAPK signaling pathway - plant	1.454	0.004	52	500
	ko00073	Cutin, suberine and wax biosynthesis	2.037	0.006	15	103

### Identification of DEGs involved in signaling pathways of grain size

**Figure 3:**
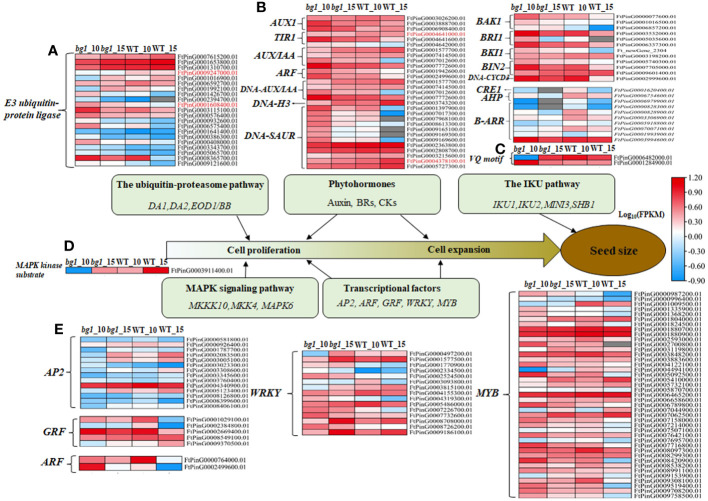
Heat map diagram of DEGs in the major signaling pathways of grain size control (Li and Li, 2016). **(A)** Ubiquitin-proteasome pathway. **(B)** Phytohormones (Auxin, Brs, CK). **(C)** IKU pathway. **(D)** MAPK signaling pathway. **(E)** Transcriptional factors.

Through the analysis of various pathways related to grain size development (**Supplementary Table 3**), there were 21 DEGs in the ubiquitin-proteasome pathway with 7 up-regulated genes and 14 down-regulated genes. Among these, two DEGs (*FtPinG0001608400.01* and *FtPinG0009247000.01*) encoding E3 ubiquitin-protein ligases was significantly different expressions in both periods between *bg1* and WT (**Figure 3A**). In the plant hormone transduction pathway, there were 29, 12 and 10 DEGs in auxin, BR and CTK metabolic pathways, respectively (**Figure 3B**). Among these, *FtPinG0004641000.01* (*TIR1*) and *FtPinG0004378100.01* (*SAUR32*) were significantly down-regulated in both periods of *bg1*. Two DEG (*VQ motif*) in the IKU pathway and one DEG (*MAPK*) in MAPK signaling pathway was detected (**Figures 3C, D**). A total of 77 DEGs were screened out from five TF families, including 14 *AP2*,5 *GRF*, 2 *ARF*, 15 *WRKY* and 41 *MYB*, which could be regulate grain size (**Figure 3E**).

### DEGs analysis of quality-related significantly enrichment pathways

**Figure 4:**
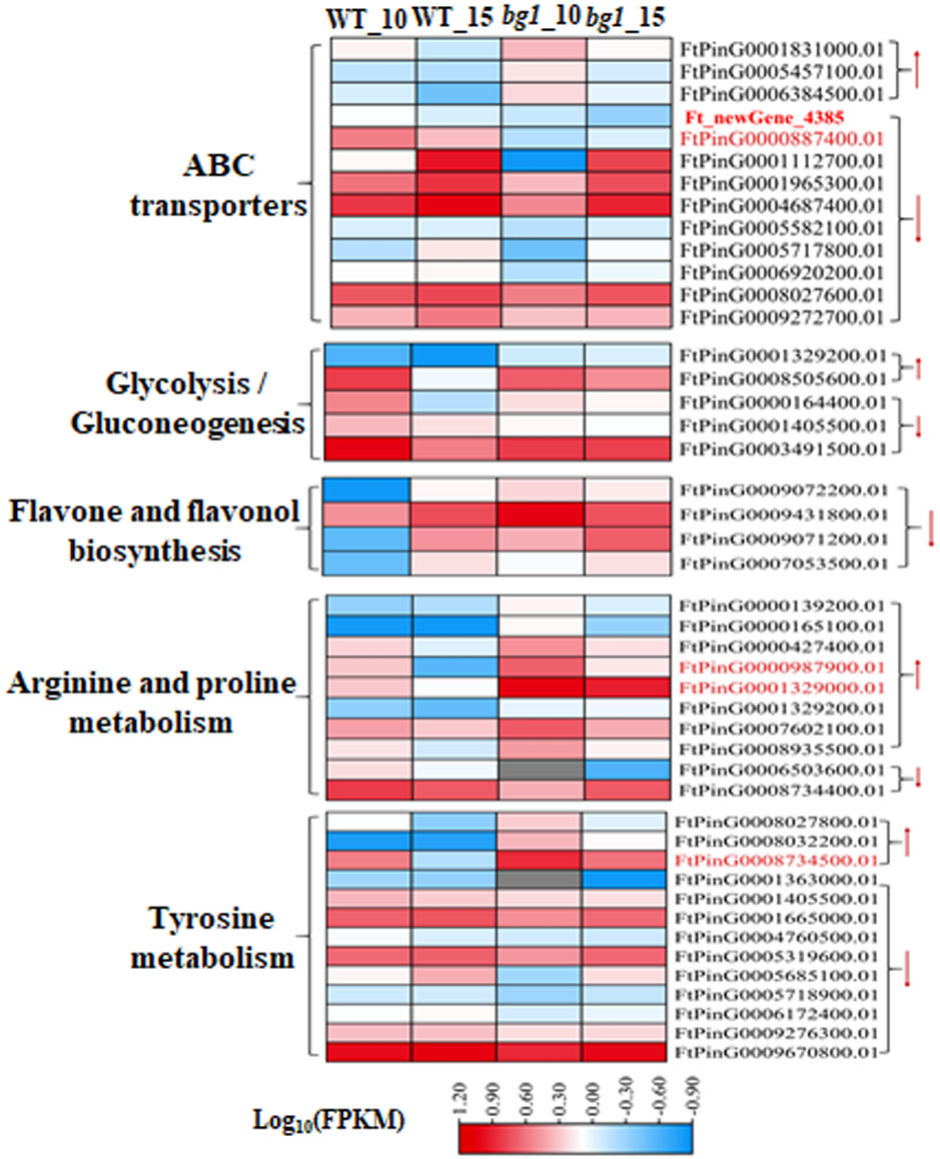
Heat map diagram of DEGs involved in quality-related pathways.

KEGG functional enrichment analysis showed that several quality-related pathways, such as ABC transporter, glycolysis/glucose production, metabolism of flavonoids and flavonol, metabolism of arginine and proline and metabolism of tyrosine were significantly enriched by DEGs. Thirteen DEGs were detected in ABC transporter pathway, among which two DEGs *Fagopyrum_tataricum_newGene_4385* (*ABC transporter C family member 9*, *ABCC9*) and *FtPinG0000887400.01* (*SRG1*) had significantly down-regulated expression in both periods of *bg1*. Five and four DEGs were detected in glycolysis/glucose production pathway and flavonoids and flavonols metabolic pathways. A total of 10 DEGs were detected in arginine and proline metabolic pathways, and *FtPinG0000987900.01* (*Aldehyde dehydrogenase family 3 member F1, ALDH3F1*) and *FtPinG0001329000.01* (*Aldehyde dehydrogenase family 2 member B7*, *ALDH2B7*) were significantly up-regulated at 10DPA and 15DPA of *bg1*. 13 DEGs were detected in the tyrosine metabolism pathway, and *FtPinG0008734500.01* (*alcohol dehydrogenase-like 1*, *ADH1*) was significantly up-regulated at 10DPA and 15DPA of *bg1* (**Figure 4**; **Supplementary Table 4**).

### Weighted gene co-expression network analysis

**Figure 5:**
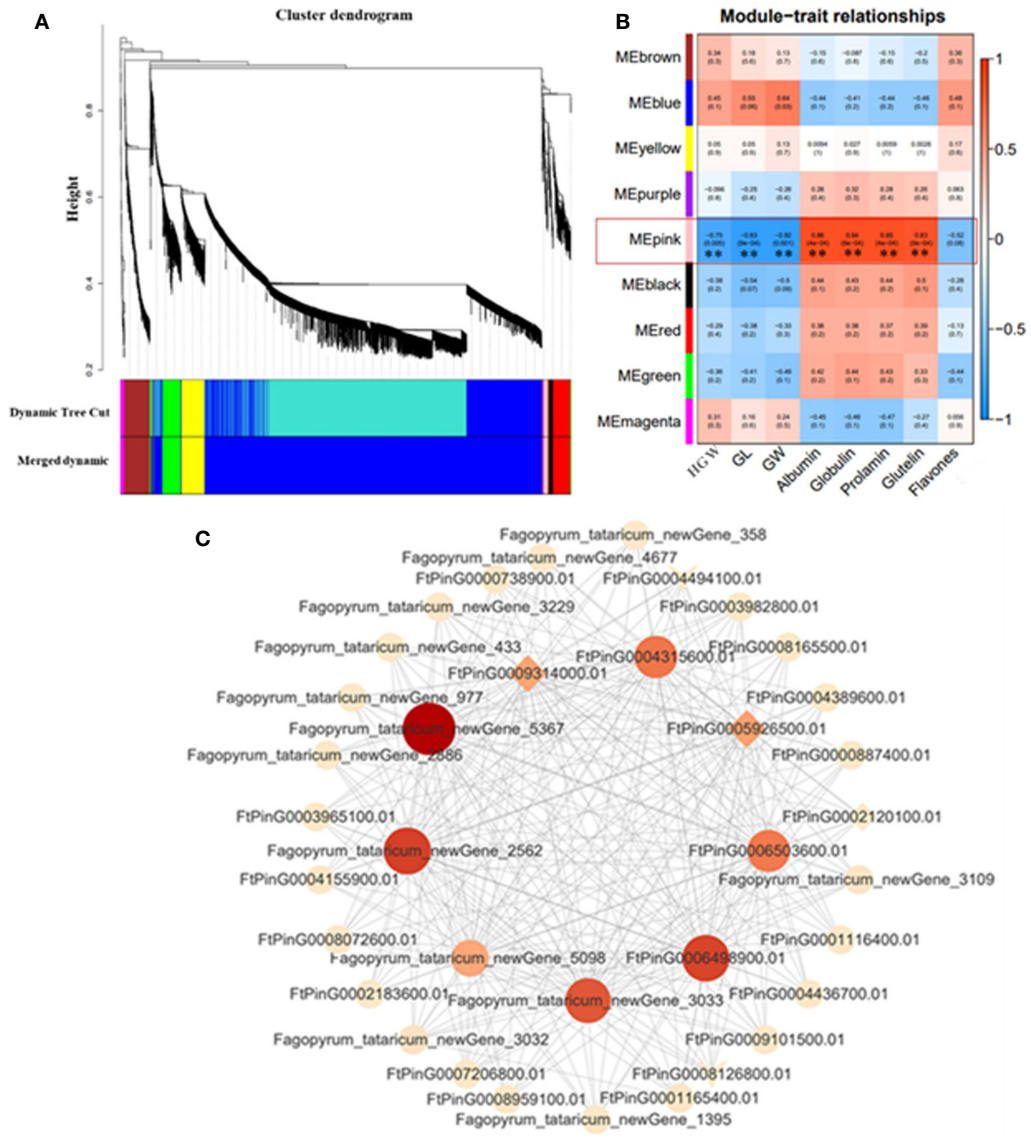
WGCNA co-expression network and modular trait correlation analysis. **(A)** Gene cluster dendrograms and module detecting. **(B)** Association analysis of gene co-expression network modules with traits. **(C)** Gene co-expression network and core genes in MEpink module.

In order to understand the gene expression regulatory network related to grain size of Tartary buckwheat, WGCNA was used to analyze all genes in 10 DPA and 15 DPA grain of *bg1* and WT, and a total of nine expression modules were obtained (**Figure 5A**). The correlation analysis of these nine expression modules with yield-related traits (Hundred-grain weight, grain length and grain width) and quality (flavonoid and protein components) was conducted (**Figure 5B**). Except for flavonoids, all the other traits were highly significantly correlated with MEpink. Cytoscape software was used to map the gene co-expression network of the top 20 MM (Module Membership) genes in MEpink Module (**Figure 5C**). Among them, 9 core genes showed high correlation with other genes (**Supplementary Table 5**), indicating that they may be the key genes for the big grain of mutant *bg1*.

### Conjoint analyses of transcriptome and metabolome sequencing

**Figure 6:**
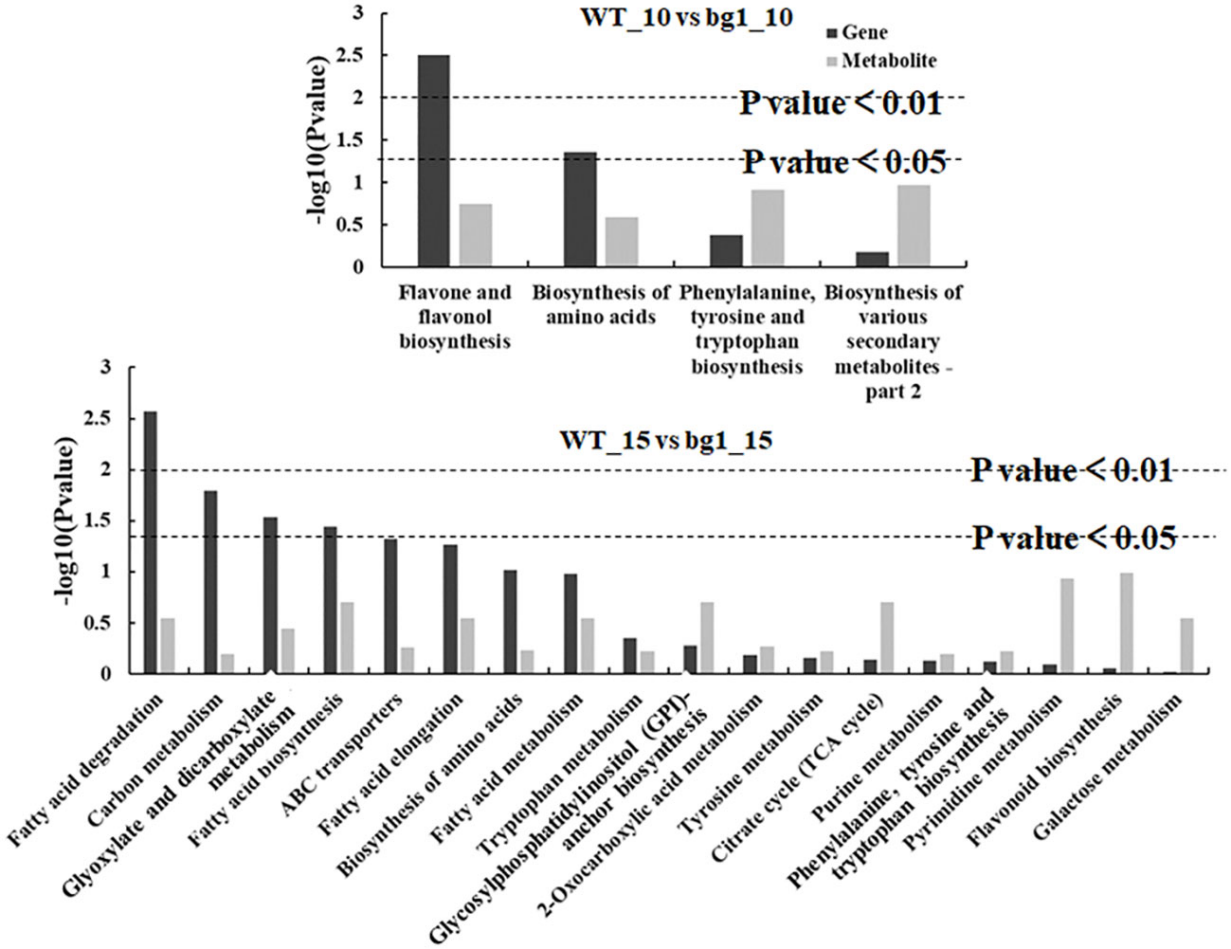
Pathways annotated with differential metabolites and differential genes.

Metabolome sequencing was carried out at 10 and 15 DPA grains of *bg1* and WT for combined with transcriptome to mine the key DEGs controlling grain size. Combined analysis of transcriptome and metabolome sequencing showed that common pathway analysis was conducted for differential genes and differential metabolites. At 10 DPA, there were 4 co-annotated metabolic pathways of differential genes and differential metabolites, and at 15 DPA, there were 18 co-annotated metabolic pathways of differential genes and differential metabolites (**Figure 6**). Among these, two metabolic pathways were simultaneously enriched at both 10 DPA and 15 DPA, namely, amino acids biosynthesis (ko01230) and phenylalanine, tyrosine and tryptophan biosynthesis (ko00400).

**Figure 7:**
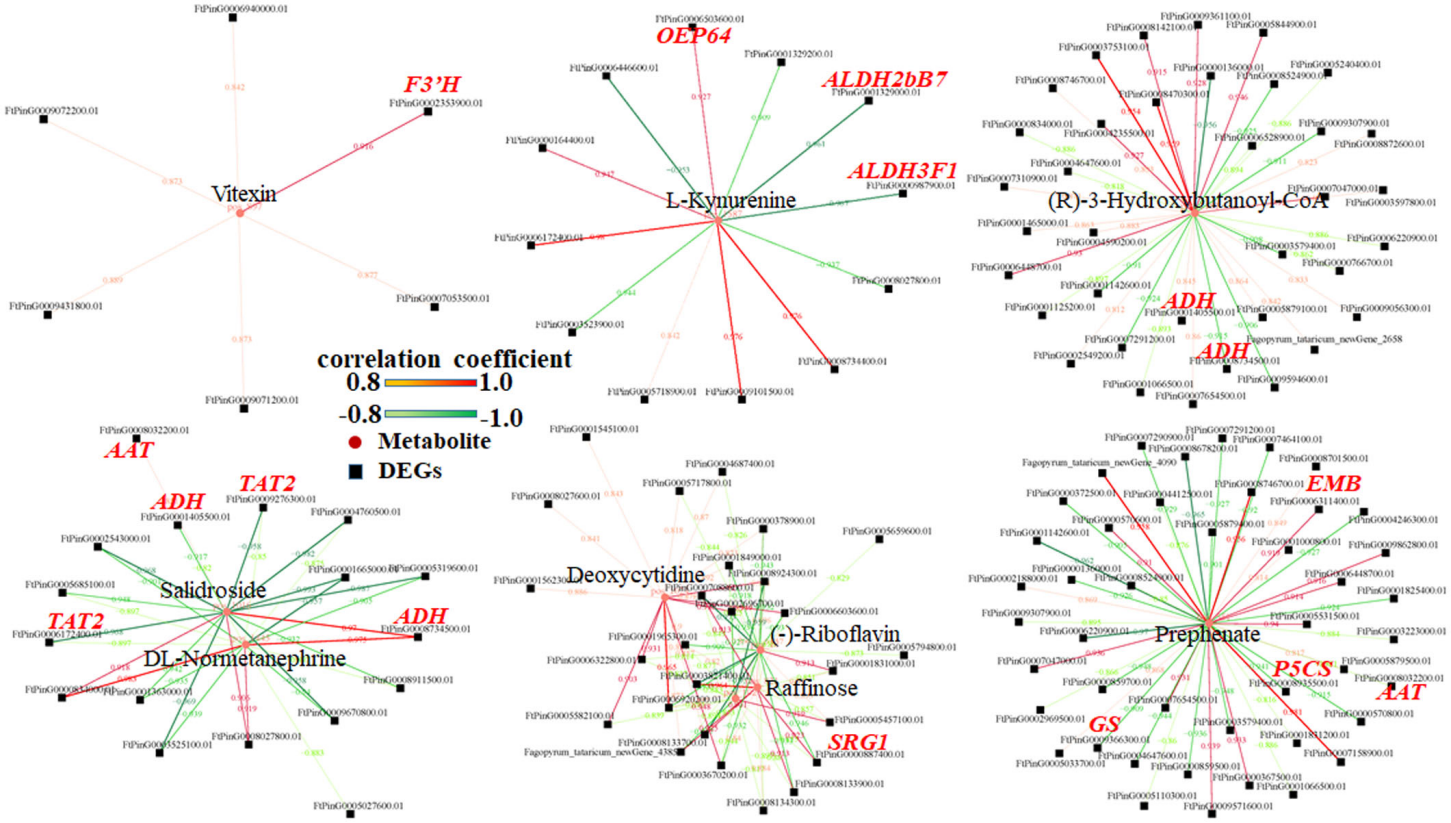
The correlation network diagram of DEGs and metabolites.

According to Pearson’s correlation coefficient, the correlation analysis between differential metabolites and differential expression genes was performed. 395 differential genes and 55 metabolites with an absolute correlation coefficient greater than 0.8 were screened out (**Supplementary Table 6**), and the correlation network diagram of DEGs and metabolite abundance was drawn (**Figure 7**).

### Screening and qRT-PCR verifying candidate genes related to grain size

Based on the above analysis, including 93 significantly up-regulated differential genes and 85 significantly down-regulated genes in both stages, DEGs involved in signaling pathways of grain size and quality-related significant enrichment pathways, WGCNA and conjoint analyses of transcriptome and metabolome sequencing, 24 candidate genes were screened out, which were presented in a variety of analysis results and had high expression level (FPKM > 10) (**Table 3**).

**Table 3:** Expression and functional annotation of candidate genes.

Gene ID	Genename	FPKM		*bg1*_10 vs WT_10		FPKM		*bg1*_15 vs WT_15		
		*bg1*_10	WT_10	log2FC	regulated	*bg1*_15	WT_15	log2FC	regulated	NR_annotation
*FtPinG0001608400.01*	*HIP1*	15.14	4.92	1.3	up	7.81	2.35	1.18	up	E3 ubiquitin-protein ligase MBR2
*FtPinG0008315000.01*	*CYP78A4*	135.92	26.99	2.04	up	50.16	1.6	2.7	up	hypothetical protein DKX38_000385
*FtPinG0007049900.01*	*CYP77A2*	119.32	37.36	1.4	up	25.58	1.77	2.12	up	cytochrome P450 77A2
*FtPinG0000987900.01*	*ALDH3F1*	58.29	14.77	1.69	up	9.36	0.58	2.78	up	Aldehyde dehydrogenase family 3
*FtPinG0008734500.01*	*ADH*	159.25	38.26	1.77	up	46.46	1.11	2.79	up	alcohol dehydrogenase-like 1
*FtPinG0001329000.01*	*ALDH2B7*	227.64	14.59	3.63	up	151.76	6.36	4	up	hypothetical protein CMV_005481
*FtPinG0009366300.01*	*GS*	44.17	16.04	1.19	up	15.87	1.57	1.97	up	glutamine synthetase leaf isozyme
*Ft_newGene_5416*	*–*	18.58	1.96	3.05	up	14.24	2.27	2.09	up	hypothetical protein FNV43_RR23572
*FtPinG0006311400.01*	*EMB*	28.66	96.86	-2.02	down	26.95	48.1	-1.09	down	hypothetical protein F0562_029405
*FtPinG0005926500.01*	*FER*	0.02	10.58	-7.75	down	0.04	4.96	-3.01	down	receptor-like protein kinase FERONIA
*FtPinG0009314000.01*	*FER*	0.45	11.11	-4.71	down	0.49	4.63	-2.29	down	receptor-like protein kinase FERONIA
*FtPinG0006498900.01*	*–*	0.1	9.98	-5.28	down	0.17	4.22	-2.67	down	–
*FtPinG0004378100.01*	*SAUR32*	2.81	6.8	-1.43	down	4.35	17.35	-1.77	down	hypothetical protein EZV62_024417
*FtPinG0001009500.01*	*MYB1*	0.25	21.46	-5.35	down	0.43	7.72	-2.83	down	Transcription repressor like
*FtPinG0002499600.01*	*ARF6*	13.21	3.86	1.49	up	2.99	0.7	–	–	auxin response factor 6 isoform X1
*FtPinG0000887400.01*	*SRG1*	0.9	19.4	-4.52	down	1.62	7.64	-2.26	down	protein SRG1-like
*FtPinG0009272700.01*	*ABCG1*	7.09	8.81	–	–	8.59	20.52	-1.49	down	ABC transporter G family member 6-like
*FtPinG0001405500.01*	*ADH*	8.14	14.77	-1.12	down	7.5	10.1	–	–	alcohol dehydrogenase-like 7
*FtPinG0002353900.01*	*CYP75B2*	49.46	98.88	-1.27	down	37.61	16.02	–	–	flavonoid 3’-hydroxylase, partial
*FtPinG0008935500.01*	*P5CS*	26.67	9.74	1.17	up	8	3.48	–	–	delta-1-pyrroline-5-carboxylate synthase
*FtPinG0006503600.01*	*OEP64*	0	10.96	-6.25	down	0.5	5.63	–	–	outer envelope protein 64
*FtPinG0008032200.01*	*AAT*	15.03	0.07	5.63	up	4.76	0.09	–	–	bifunctional aspartate aminotransferase
*FtPinG0005685100.01*	*NAAT1*	0.82	5.18	-2.15	down	7.73	17.46	–	–	probable aminotransferase TAT2
*FtPinG0009276300.01*	*NAAT1*	7.76	13.21	-1.03	down	8.48	12.48	–	–	probable aminotransferase TAT2

All expression data are given as mean of FPKM with three individual biological replications.

Correlation analysis was conducted between FPKM values of candidate genes and yield and quality-related traits of mutant *bg1* and WT (**Table 4**). Among these candidate genes, most genes were significantly or highly significantly associated with grain traits, especially grain width. Besides, several genes were significantly or highly significantly correlated with yield and quality traits, simultaneously, including *FtPinG0001608400.01* (*HIP1*) detected in the ubiquitin-proteome pathway, *FtPinG0001329000.01* (*ALDH2B7*) detected in arginine and proline metabolic pathways and conjoint analyses of transcriptome and metabolome sequencing, *Ft_newGene_5416* screened out by conjoint analyses of transcriptome and metabolome sequencing, *FtPinG0005926500.01* (*FER*), *FtPinG0009314000.01*(*FER*) and *FtPinG0006498900.01* screened out by WGCNA analysis, *FtPinG0004378100.01* (*SAUR32*) detected in auxin metabolic pathways, *FtPinG0000887400.01* (*SRG1*) detected in ABC transporter pathway and conjoint analyses.

**Table 4:** Correlation analysis of candidate gene expression with yield and quality traits.

Gene ID	TGW	LW	GL	GW	Flavones	Albumin	Globulin	prolamin	Glutelin
*FtPinG0001608400.01*	0.689*	-0.803**	0.781**	0.859**	0.667*	-.698*	-0.649*	-0.685*	-0.662*
*FtPinG0008315000.01*	0.616*	-0.732**	0.715**	0.789**	0.579*	-.622*	-0.586*	-0.617*	-0.622*
*FtPinG0007049900.01*	0.484	-0.701*	0.661*	0.743**	0.449	-0.53	-0.495	-0.529	-0.514
*FtPinG0000987900.01*	0.442	-0.726**	0.641*	0.747**	0.483	-0.539	-0.527	-0.532	-0.538
*FtPinG0008734500.01*	0.557	-0.732**	0.697*	0.780**	0.514	-.592*	-0.553	-0.590*	-0.588*
*FtPinG0001329000.01*	0.809**	-0.827**	0.900**	0.941**	0.710**	-.865**	-0.828**	-0.858**	-0.837**
*FtPinG0009366300.01*	0.554	-0.712**	0.668*	0.751**	0.527	-0.562	-0.526	-0.561	-0.55
*Ft_newGene_5416*	0.794**	-0.849**	0.918**	0.964**	0.700*	-.948**	-0.933**	-0.940**	-0.909**
*FtPinG0006311400.01*	-0.644*	0.541	-0.718**	-0.695*	-0.375	0.771**	0.750**	0.768**	0.781**
*FtPinG0005926500.01*	-0.722**	0.665*	-0.828**	-0.819**	-0.565	0.856**	0.868**	0.857**	0.802**
*FtPinG0009314000.01*	-0.724**	0.652*	-0.803**	-0.796**	-0.51	0.838**	0.815**	0.831**	0.796**
*FtPinG0006498900.01*	-0.760**	0.674*	-0.816**	-0.811**	-0.526	0.835**	0.789**	0.822**	0.817**
*FtPinG0004378100.01*	-0.595*	0.669*	-0.692*	-0.736**	-0.612*	0.724**	0.710**	0.729**	0.589*
*FtPinG0001009500.01*	-0.584*	0.498	-0.625*	-0.622*	-0.318	0.695*	0.692*	0.694*	0.815**
*FtPinG0002499600.01*	0.481	-0.710**	0.659*	0.747**	0.515	-0.522	-0.49	-0.515	-0.518
*FtPinG0000887400.01*	-0.688*	0.641*	-0.774**	-0.772**	-0.528	0.780**	0.806**	0.783**	0.654*
*FtPinG0009272700.01*	-0.456	0.590*	-0.541	-0.604*	-0.489	0.590*	0.632*	0.602*	0.508
*FtPinG0001405500.01*	-0.462	0.384	-0.607*	-0.556	-0.311	0.703*	0.714**	0.693*	0.645*
*FtPinG0002353900.01*	-0.164	0.048	-0.196	-0.143	0.066	0.235	0.241	0.235	0.237
*FtPinG0008935500.01*	0.461	-0.705*	0.647*	0.740**	0.53	-0.534	-0.52	-0.526	-0.533
*FtPinG0006503600.01*	-0.816**	0.745**	-0.854**	-0.868**	-0.557	0.886**	0.849**	0.878**	0.849**
*FtPinG0008032200.01*	0.719**	-0.798**	0.729**	0.820**	0.238	-.770**	-0.747**	-0.783**	-0.668*
*FtPinG0005685100.01*	-0.457	0.608*	-0.519	-0.610*	-0.5	0.499	0.479	0.504	0.484
*FtPinG0009276300.01*	-0.552	.595*	-.671*	-0.688*	-0.438	.681*	0.757**	0.710**	0.552

*P ≤ 0.05, **P ≤ 0.01, “-” means negative correlation or positive correlation.

**Figure 8:**
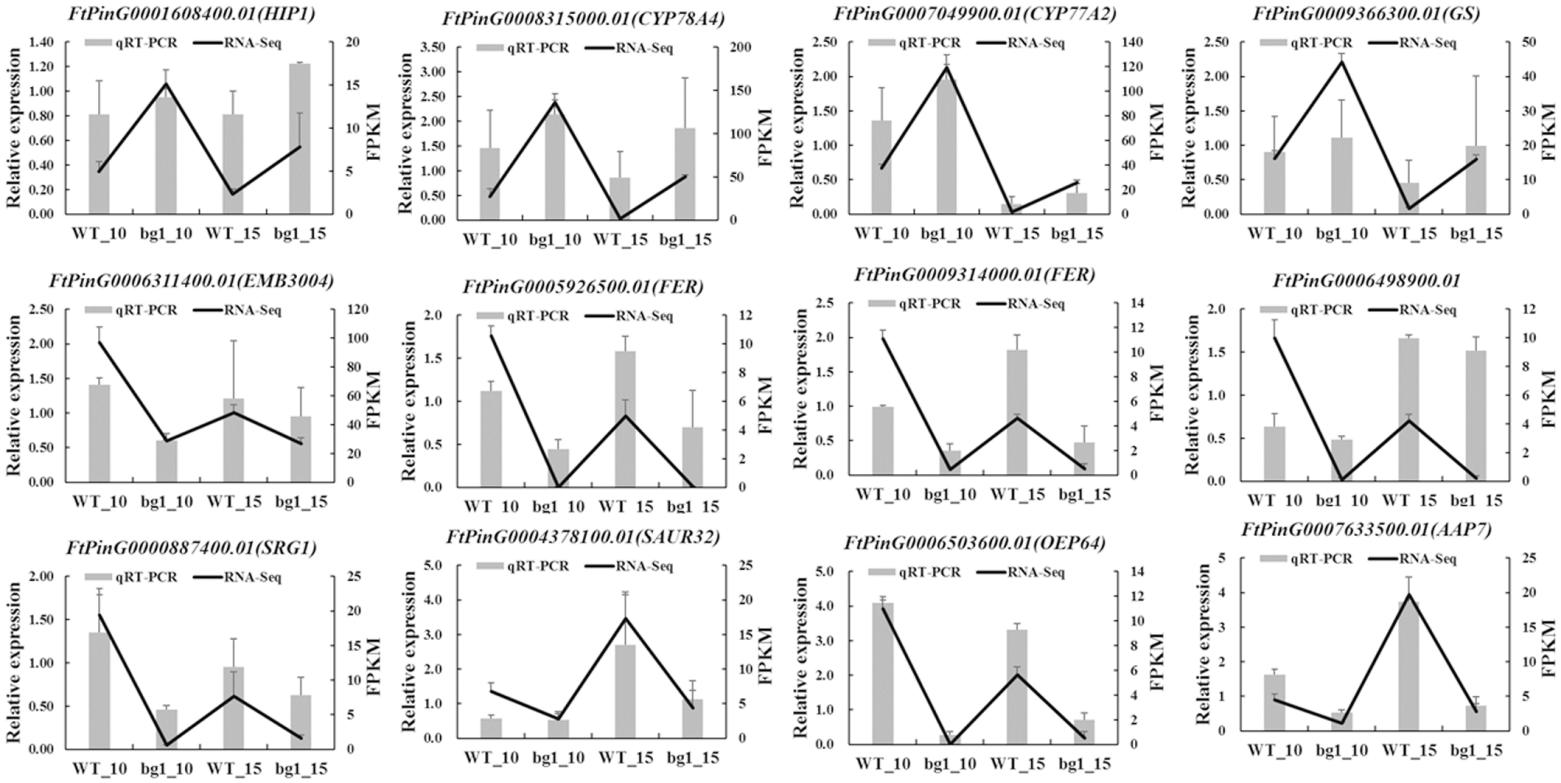
qRT-PCR validation of 12 differentially expressed genes related to grain size. The column diagram represents the relative expression determined with qRT-PCR and the line chart represents the level of expression (FPKM) determined with RNA-seq. Error bars indicate the standard errors from three independent biological and three technical replicates for qRT-PCR data.

To validate the expression patterns of DEGs obtained from RNA-Seq analysis, qRT-PCR was conducted to examine the expression levels of 12 DEGs in 10 DPA and 15 DPA of WT and *bg1*. Details of the primers used for the qRT-PCR assay were listed in **Supplemental Table 7**. The expression levels of these selected DEGs from qRT-PCR analyses were generally consistent with FPKM values deduced from RNA-Seq (**Figure 8**). These results confirm the reliability of the transcriptomic profiling data estimated from RNA-Seq data.

## Discussion

Major breeding objectives for Tartary buckwheat included developing shorter plants to prevent lodging and creating new high-yielding varieties that produce grains with big grains and excellent quality. Improvement of Tartary buckwheat has previously been achieved through the introduction of landraces and selections of natural or artificial mutants in the past (Zhang et al., 2017). Through large-scale screening of genetic resources and EMS mutagenized lines, Suzuki et al., 2014a; Suzuki et al., 2014b) developed a new Tartary buckwheat cultivar ‘‘Manten-Kirari’’ that showed rutin hydrolysis by rutinosidase as the major factor leading to bitterness. In this study, we obtain a big-grain mutant with high flavonoid content by EMS mutagenesis, which is an excellent breeding resource with high-yield and high quality, and it is also an excellent resource for studying grain yield and quality traits because of its simple genetic background compared to WT.

Various agronomic traits have been subjected to artificial or natural selection during crop domestication, and understanding the underlying molecular mechanisms will facilitate genetic improvement of these traits and the breeding of elite crop varieties. For example, *PROG1* (*PROSTRATE GROWTH 1*), *GIF1* (*GRAIN INCOMPLETE FILLING 1*) and *Sh3/sh4* during domestication resulted in the erect growth habit, non-shattering phenotype, increased grain number, and improved grain production of modern rice cultivars, respectively (Li et al., 2006; Tan et al., 2008; Wang et al., 2008). *Teosinte branched1* (*tb1*) led to the enhance of apical dominance in all modern maize cultivars (Dong et al., 2017). Tartary buckwheat is an important cash crop, and is one of the main ways to obtain economic benefits for farmers in high-cold mountainous districts. Grains are the most important harvest trait because grain size/weight directly determines Tartary buckwheat yield, and selection for large grains has been an important target during Tartary buckwheat domestication and production. While the molecular mechanisms of grain development are poorly understood in *Fagopyrum*. In this study, we used transcriptome and metabolome sequencing, and conjoint analyses at different development stages grains of a big-grain mutant (*bg1*) and WT to mine the key genes controlling grain size and provide theoretical foundations of genetic improvement in Tartary buckwheat.

An increasing number of studies have confirmed that RNA-seq is a powerful method to investigate the transcriptome profiles and mine key candidate genes controlling many agronomically important traits in Tartary buckwheat (Logacheva et al., 2011; Huang et al., 2017; Gao et al., 2017; Wu et al., 2017; Yao et al., 2017). In this study, we performed genome-wide transcriptome sequencing using 10 DPA and 15 DPA grains of *bg1*, and identified 4108 DEGs including 93 significantly up-regulated differential genes and 85 significantly down-regulated genes in both stages, simultaneously. Among these, *FtPinG0008315000.01* (*CYP78A4*) and *FtPinG0007049900.01* (*CYP77A2*) had high expression level in *bg1* and significantly up-regulated expression than WT, which had been reported to regulated grain size in Arabidopsis and rice (Adamski et al., 2009; Fang et al., 2012; Yang et al., 2013; Xu et al., 2015; Maeda et al., 2019). Meanwhile, we identified DEGs involved in several signaling pathways of grain size (Li and Li, 2017), such as ubiquitin-proteasome pathway, IKU pathway, MAPK signaling pathway, plant hormone transduction pathway (auxin, BR and CK) and five TF families, including AP2, GRF, ARF, WRKY and MYB. Besides, there were significant differences not only in grain size but also in flavonoid and protein contents between *bg1* and WT. Based on the KEGG enrichment analysis for DEGs, we identified DEGs in several quality-related pathways, such as ABC transporter, glycolysis/glucose production, metabolism of flavonoids and flavonol, metabolism of arginine and proline and metabolism of tyrosine, which were significantly enriched by DEGs. *FtPinG0000987900.01*(*ALDH3F1*)and *FtPinG0001329000.01*(*ALDH2B7*)had simultaneously significantly up-regulated expression in 10 DPA and 15 DPA of *bg1*, belong to ALDH family members and may be involved in abiotic stress (Skibbe et al., 2002), which showed *bg1* may be more resistant to stress than WT. All of these DEGs could be the key genes controlling grain size and quality of Tartary buckwheat, and provided important basic expression data for the mechanism study of yield and quality traits of Tartary buckwheat.

WGCNA is an important method for rapidly mining key genes that are highly correlated with traits from multiple transcriptome data (Zhang and Horvath, 2005). The combination of transcriptome data and WGCNA algorithm to study the core genes related to plant growth and development has been widely used in the research of the morphogenesis and development regulation mechanism of plant organs such as flowers, leaves and fruits, and the function prediction of unknown genes (Hollender et al., 2014; Greenham et al., 2017; Luo et al., 2019; Sun et al., 2019; Kuang et al., 2021). In this study, we performed WGCNA for all genes in 10 DPA grains and 15 DPA grains of *bg1* and WT, and obtained nine expression modules, among which MEpink had high correlation with yield and quality-related traits. In MEpink module, the gene co-expression network was conducted and 9 core genes showed high correlation with other genes. Among these, two plasma membrane receptor kinase FERONIA (*FtPinG0005926500.01* and *FtPinG0009314000.01*, *FER*) had simultaneously significantly down-regulated expression in 10 DPA and 15 DPA of *bg1*, which had been reported for controlling cell elongation and hormone crosstalk as an important regulatory node (Guo et al., 2009; Deslauriers and Larsen, 2010; Duan et al., 2010; Yu et al., 2012), and negatively regulated grain size of *Arabidopsis thaliana* (Yu et al., 2014).

In this study, untargeted LC-MS were performed and and conjoint analyses of transcriptome and metabolome sequencing identified 394 DEGs, which had high correlation with metabolites. Among these genes, three genes (*FtPinG0001329000.01*, *FtPinG0009366300.01* and *Ft_newGene_5416*) had simultaneously significantly up-regulated expression and three genes (*FtPinG0006311400.01*, *FtPinG0000887400.01* and *FtPinG0009272700.01*) had simultaneously down-regulated expression in 10 DPA and 15 DPA of *bg1. FtPinG0009366300.01* (Glutamine synthetase, *GS*) had up-regualted expression in *bg1* and encoded a key enzyme in the formation of the amino acid glutamine during N assimilation, which had been reported to involved in the control of grain production in rice (Tabuchi et al., 2005; Gaur et al., 2012), maize (Martin et al., 2006) and wheat (Guo et al., 2013). *SRG1*, encoding a kinesin-4 protein, is an important factor for grain shape by controlling grain width through cell proliferation (Qin et al., 2018). In this study, *FtPinG0000887400.01* (*SRG1*) detected in ABC transporter pathway negatively regulated grain size of *bg1*, similar to rice (Qin et al., 2018).

In conclusion, we generated transcriptome and metabolome sequencing from developing grains of big grain mutant *bg1* and WT at 10 and 15 DPA, and also performed comprehensive transcriptomic analysis, including DGEA, GO term and KEGG pathway enrichment, identification of DEGs involved in signaling pathways of grain size and quality-related significant enrichment pathways, WGCNA and conjoint analyses of transcriptome and metabolome sequencing. Twenty-four potential candidate genes were screened out and correlation analysis of candidate gene expression with yield and quality traits was performed. The data generated here will be an invaluable resource for the genetic dissection of Tartary buckwheat yield and quality-related traits, and our results provide additional insights into the identification and functions of causal candidate genes responsible for the variation in grain size/weight in Tartary buckwheat.

## Data availability statement

All raw sequences for transcriptome are available in the NCBI Sequence Read Archive under Bioproject # PRJNA91107.

## Author contributions

ZY conceived, supervised the experiment, and revised the manuscript. XF analyzed the data and wrote the draft manuscript. YQW, JC and LY performed research. CL, JZ and MD assisted in editing the manuscript. AJ, JL, YCW and XH managed field research and plant propagation. All authors contributed to the article and approved the submitted version.
